# Aldose reductase modulates acute activation of mesenchymal markers via the β-catenin pathway during cardiac ischemia-reperfusion

**DOI:** 10.1371/journal.pone.0188981

**Published:** 2017-11-30

**Authors:** Devi Thiagarajan, Karen O’ Shea, Gopalkrishna Sreejit, Radha Ananthakrishnan, Nosirudeen Quadri, Qing Li, Ann Marie Schmidt, Kenneth Gabbay, Ravichandran Ramasamy

**Affiliations:** 1 Diabetes Research Program, Department of Medicine, New York University Langone Medical Center, New York, New York, United States of America; 2 Department of Pediatrics, Children’s Nutrition Research Center, Baylor College of Medicine, Houston, Texas, United States of America; Virginia Commonwealth University Medical Center, UNITED STATES

## Abstract

Aldose reductase (AR: human, AKR1B1; mouse, AKR1B3), the first enzyme in the polyol pathway, plays a key role in mediating myocardial ischemia/reperfusion (I/R) injury. In earlier studies, using transgenic mice broadly expressing human *AKR1B1* to human-relevant levels, mice devoid of *Akr1b3*, and pharmacological inhibitors of AR, we demonstrated that AR is an important component of myocardial I/R injury and that inhibition of this enzyme protects the heart from I/R injury. In this study, our objective was to investigate if AR modulates the β-catenin pathway and consequent activation of mesenchymal markers during I/R in the heart. To test this premise, we used two different experimental models: *in viv*o, *Akr1b3* null mice and wild type C57BL/6 mice (WT) were exposed to acute occlusion of the left anterior descending coronary artery (LAD) followed by recovery for 48 hours or 28 days, and *ex-vivo*, WT and *Akr1b3* null murine hearts were perfused using the Langendorff technique (LT) and subjected to 30 min of global (zero-flow) ischemia followed by 60 min of reperfusion. Our *in vivo* results reveal reduced infarct size and improved functional recovery at 48 hours in mice devoid *of Akr1b3* compared to WT mice. We demonstrate that the cardioprotection observed in *Akr1b3* null mice was linked to acute activation of the β-catenin pathway and consequent activation of mesenchymal markers and genes linked to fibrotic remodeling. The increased activity of the β-catenin pathway at 48 hours of recovery post-LAD was not observed at 28 days post-infarction, thus indicating that the observed increase in β-catenin activity was transient in the mice hearts devoid of *Akr1b3*. In *ex vivo* studies, inhibition of β-catenin blocked the cardioprotection observed in *Akr1b3* null mice hearts. Taken together, these data indicate that AR suppresses acute activation of β-catenin and, thereby, blocks consequent induction of mesenchymal markers during early reperfusion after myocardial ischemia. Inhibition of AR might provide a therapeutic opportunity to optimize cardiac remodeling after I/R injury.

## Introduction

Acute myocardial infarction (AMI) remains the leading cause of morbidity and mortality worldwide [1]. The extent of myocardial tissue loss (infarct size) is a key determinant of the prognosis of patients with AMI. Timely reperfusion is the most effective way to limit infarct size in patients with AMI [2]. However, efficacy of reperfusion therapy is impaired by factors such as the severity of ischemia, inadequate reflow, presence of residual stenosis, coronary reocclusion, and reperfusion injury [3–5]. In the quest for novel therapeutic strategies for acute myocardial ischemia/reperfusion (I/R) injury, we have focused on interventions that modulate substrate metabolism [6, 7]. In this context, we and others demonstrated that the aldose reductase (AR) pathway contributes to myocardial I/R injury and that the inhibition of AR protects hearts from I/R damage [8–13]. Earlier studies showed that increased flux via AR during I/R leads to ATP depletion and increased mitochondrial oxidative stress, thereby significantly impeding the recovery process in the heart [9, 14, 15]. We and others demonstrated that pharmacological inhibition of AR improves functional recovery and reduces myocardial I/R injury [11, 13, 16, 17].

The Wnt/β-catenin pathway plays an important role in various biological processes including development, differentiation, proliferation and tissue homeostasis [18, 19]. Activation of the Wnt pathway culminates in the transcription of Wnt target genes via β-catenin. Wnt proteins form a family of highly conserved secreted signaling molecules. Upon binding of Wnt to the seven-transmembrane domain spanning frizzled (Fzd) receptor and the co-receptor lipoprotein receptor-related 5/6 (Lrp5/6) proteins, GSK3β is inactivated, thereby preventing the breakdown of β-catenin. After stabilization and accumulation, β-catenin enters the nucleus, where it binds to LEF/TCF transcription factors to activate the transcription of Wnt target genes [20, 21]. Several studies have shown involvement of the canonical Wnt/β-catenin signaling pathway in the pathogenesis of I/R injury [22–24] and that phosphorylation of GSK3β is a key determinant of β-catenin activation [25–27]. Since we previously demonstrated that AR alters the phosphorylation state of GSK3β during I/R [14], here we investigated if AR modulates β-catenin activity and consequent activation of mesenchymal markers during IR in the heart. We used two distinct models to study whether AR affects myocardial β-catenin and consequent activation of mesenchymal markers during I/R: a transient occlusion and reperfusion of the left anterior descending coronary artery (LAD) *in vivo* model of I/R, and an *ex vivo* intact heart preparation subjected to I/R. We employed mice devoid of AR (*Akr1b3* null mice) to determine whether altered flux via AR influences myocardial β-catenin during I/R. Our results indicate that genetic deletion *of Akr1b3* drives acute induction of mesenchymal markers, at least in part via induction of the β-catenin pathway during I/R in the heart.

## Results

### *Akr1b3* deletion reduces infarct size and improves functional recovery after I/R

**Fig 1:**
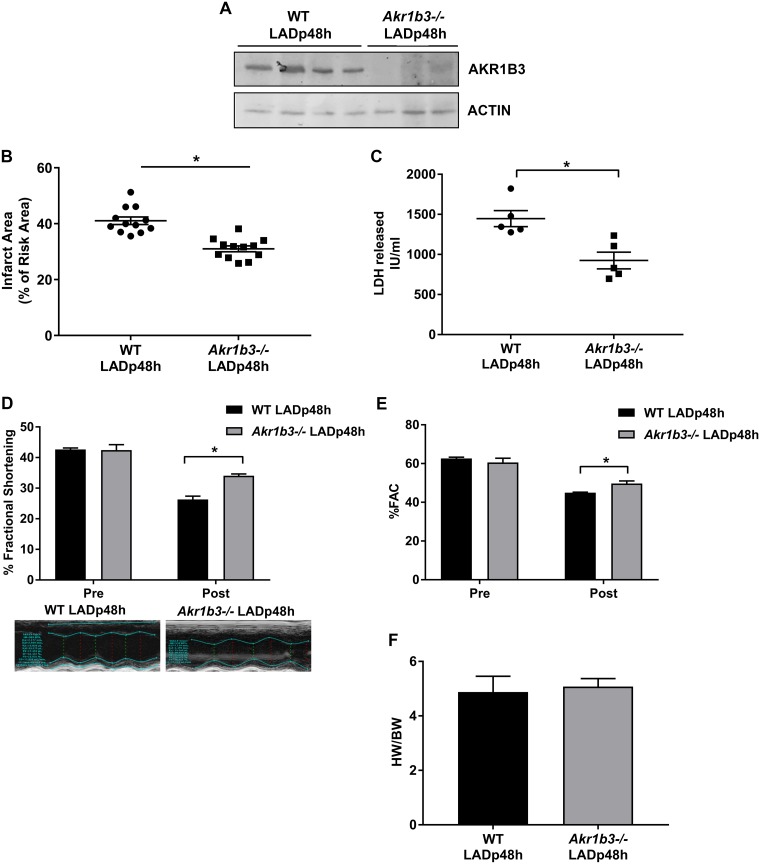
Cardioprotection in *Akr1b3* null I/R mice. Male WT and *Akr1b3* null mice were subjected to LAD occlusion followed by reperfusion at age 4 months. (A) Western blot analysis of AKR1B3 in heart tissue lysate at 48 h post-LAD was performed and normalized to levels of B-ACTIN, N = 4 mice/genotype. (B) *Akr1b3* null mice exhibit decreased infarct area (expressed as in % of infarct area/area at risk) after LAD/reperfusion vs. WT mice (n = 10/group; * p<0.05 vs. WT LAD) with no genotype differences in area at risk (data not shown). (C) Total plasma LDH levels were measured at 48 h post-LAD, N = 6 mice/group. (D) Changes in % fractional shortening (FS) with representative echocardiographic image. (E) % of fractional area change (FAC), N = 10/group. (F) The ratio of heart weight to body weight was measured, N = 10/group. Error bars represent mean ± SEM. * p<0.05, unless otherwise noted.

We subjected male WT and *Akr1b3* null mice to left anterior descending coronary artery (LAD) occlusion for 30 min, followed by 48 hours of reperfusion (LADp48h). The expression or absence of AKR1B3 in WT and *Akr1b3* null mice, respectively, was verified by Western blot (Fig 1A). Infarct size, as a percent of area at risk, measured at 48 h post I/R, was significantly lower in *Akr1b3* null mice vs. WT mice (Fig 1B), but there were no differences in area at risk between the two genotypes (data not shown). Plasma LDH levels, a marker of myocardial injury, were significantly lower in mice devoid of *Akr1b3* vs. WT mice at 48 h post I/R (Fig 1C). Echocardiographic measurements revealed significant differences in fractional shortening and fractional area change in *Akr1b3* null mice compared to WT mice (Fig 1D and 1E). Cardiac hypertrophy, assessed by measuring the heart weight to body weight ratio, revealed no significant differences in *Akr1b3* null vs WT mice hearts at 48 h post I/R (Fig 1F). Taken together, these results demonstrate improved functional recovery and reduced markers of injury in *Akr1b3* null mice hearts compared to WT mice after I/R.

### Upregulation of TGFB2 in *Akr1b3* null mice hearts after I/R

**Fig 2:**
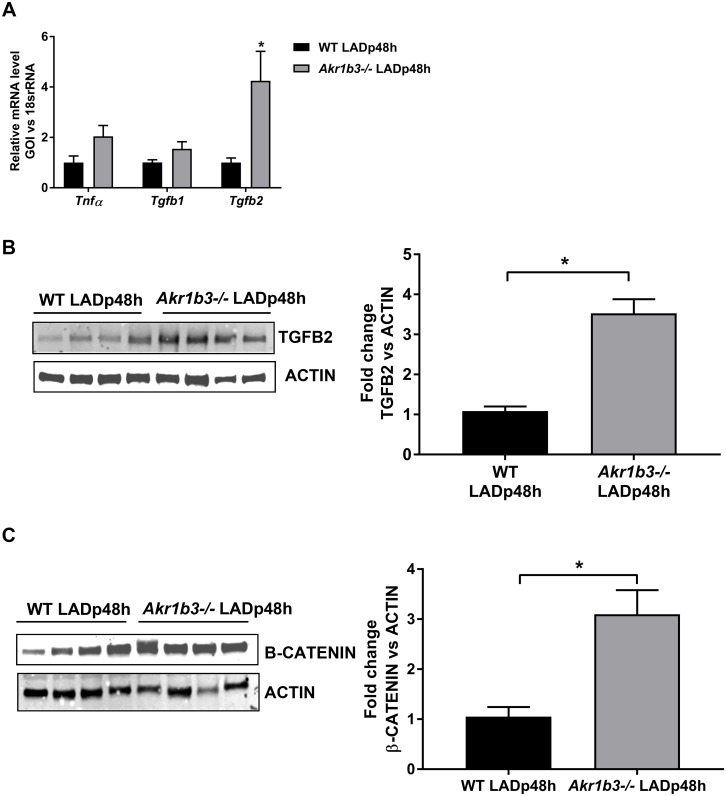
Upregulation of TGFβ2/β-catenin in *Akr1b3 null* mice 48 h post-I/R. (A) qRT-PCR on *Tnfα*, *Tgfb1* and *Tgfb2* transcripts from RNA isolated in the heart tissues, N = 5 mice/group. (B) Western blot of TGFB2 protein expression normalized to Beta-actin, N = 4 mice/group (C) Western blot of β-catenin protein expression normalized to Beta-actin, N = 4 mice/group. Error bars represent mean ± SEM. * p<0.05, unless otherwise noted.

The early phase of recovery post-LAD ligation involves an inflammatory response with the release of cytokines. Hence, we quantified the expression of prominent cytokines involved in wound healing responses, including *Tnfα*, *Tgfb1* and *Tgfb2* via qRT-PCR [28, 29]. There were no differences in expression of *Tnfa* or *Tgfb1* between the two mouse groups but we observed ≈4-fold higher *Tgfb2* mRNA levels in *Akr1b3* null vs. WT mice LADp48h mice (Fig 2A). Consistent with changes in mRNA levels, TGFB2 protein levels were upregulated by ≈3-fold in mice hearts devoid of *Akr1b3* compared to WT mice (Fig 2B). Since studies have implicated TGFB2 in modulating β-catenin expression, we examined protein expression of β-catenin in WT and *Akr1b3* null mice hearts [21, 30]. Western blot analysis of these heart tissues showed an ≈3-fold increase in β-catenin protein expression in *Akr1b3* null mice vs. WT mice post-I/R (Fig 2C). These results indicate that deletion of *Akr1b3* enhances TGFB2 and β-catenin expression after I/R in mice hearts.

### Mesenchymal activation in mice hearts devoid of *Akr1b3*

Loss of cardiomyocytes during ischemia is compensated for by the activation of existing fibroblasts or through the trans-differentiation of endothelial cells to mesenchymal cells via endothelial mesenchymal transition (EndMT) [31–33]. Several studies have implicated TGFB2 in the EndMT pathway [31–33]. Canonical Wnt signaling is involved in EndMT-mediated cardiac fibrosis and stabilized β-catenin serves as a marker for activated Wnt signaling [22, 31, 34]. Hence, we investigated whether the increase in TGFB2 and β-catenin observed in *Ak1br3* null mice hearts (Fig 2A–2C) leads to EndMT transition.

**Fig 3:**
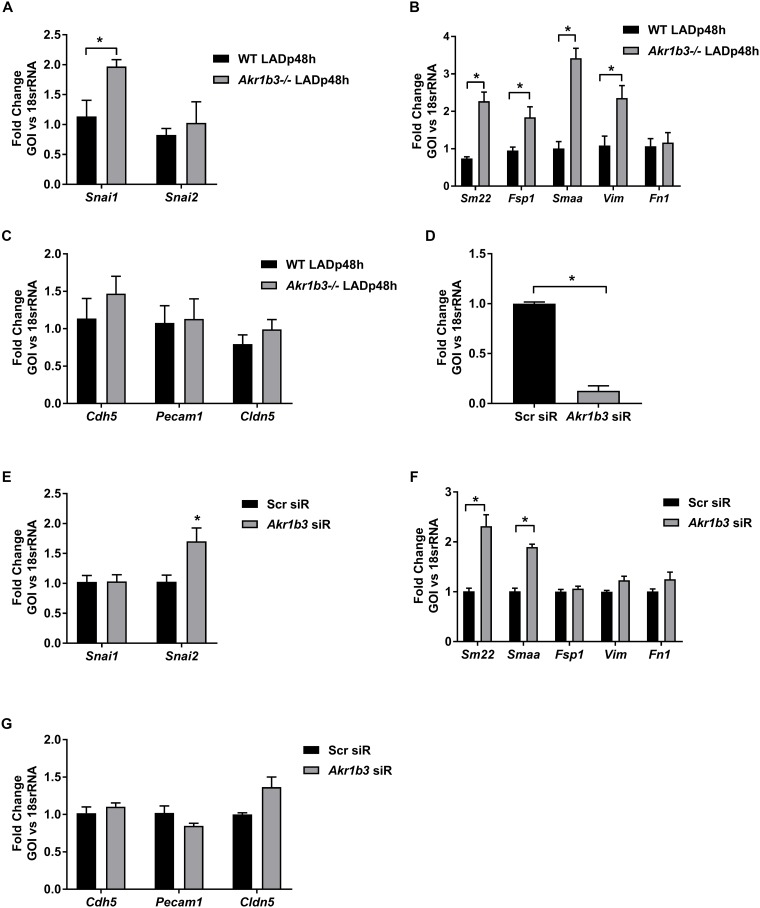
Mesenchymal activation in *Akr1b3* null mice hearts 48 h post-I/R. RNA isolated from heart tissues from mice subjected to I/R. (A) qRT-PCR on EndoMT transcription factors- *Snai1* and *Snai2*. (B) qRT-PCR on mesenchymal markers as indicated. (C) qRT-PCR on endothelial markers as indicated. MAECs transfected with scrambled and siR against *Akr1b3* and probed for (D) *Akr1b3*. € qRT-PCR on EndoMT transcription factors as indicated. (F) qRT-PCR on mesenchymal markers as indicated. (G) qRT-PCR on endothelial markers as indicated. N = 3 mice/group. Error bars represent mean ± SEM. * p<0.05, unless otherwise noted.

EndMT is characterized by reduction of endothelial markers platelet/endothelial adhesion molecule (*Pecam1*, cadherin 5 (*Cdh5) and* claudin 5 *(Cldn5*), with concomitant activation of mesenchymal markers (α-smooth muscle actin *(Smaa)*, Transgelin (*Sm22)*, *Vimentin (Vim)*, *S100a4* (*Fsp1)* and fibronectin 1 (*Fn1)* and the related EndMT transcription factors (*Snai1* and *Snai2*). Analysis of the transcription factors involved in EndMT, *Snai1* and *Snai2* revealed upregulation of *Snai1*, but not *Snai2* in hearts devoid of *Akr1b3* (Fig 3A). We observed increased expression of all mesenchymal markers, *Smaa*, *Sm22*, *Vim* and *Fsp1*, except *Fn1* (Fig 3B) in *Akr1b3* null hearts. Analysis of endothelial markers showed no differences in gene expression of *Pecam1*, *Cdh5* and *Cldn5* (Fig 3C). To confirm these results *in vitro*, we employed mouse primary aortic endothelial cells (MAEC), which were transfected with siRNA against *Akr1b3 (Abr1b3* siR). Scrambled siRNA was used as a negative control (scr siR). 90% knockdown efficiency was achieved in these cells (Fig 3D). These cells were then subjected to 30 min of hypoxia followed by 1 h reperfusion (H/R). qRT-PCR analysis showed a significant increase in EndMT transcription factor *Snai2* (Fig 3E), a significant increase in *Smaa*, but not in the other mesenchymal markers (Fig 3F) and no reduction in the endothelial markers (Fig 3G). Taken together, these results ruled out the possibility of classical EndMT processes, both *in vitro* and *in vivo*, yet distinct mesenchymal activation was observed in cells depleted of *Akr1b3*. Furthermore, increased expression of mesenchymal markers (*Smaa*, *Sm22*, *Vim*, *Fsp1*) suggested an activated fibroblast phenotype in endothelial cells derived from mice devoid of *Akr1b3*.

### Induction of genes linked to fibrosis in hearts devoid of *Akr1b3*

**Fig 4:**
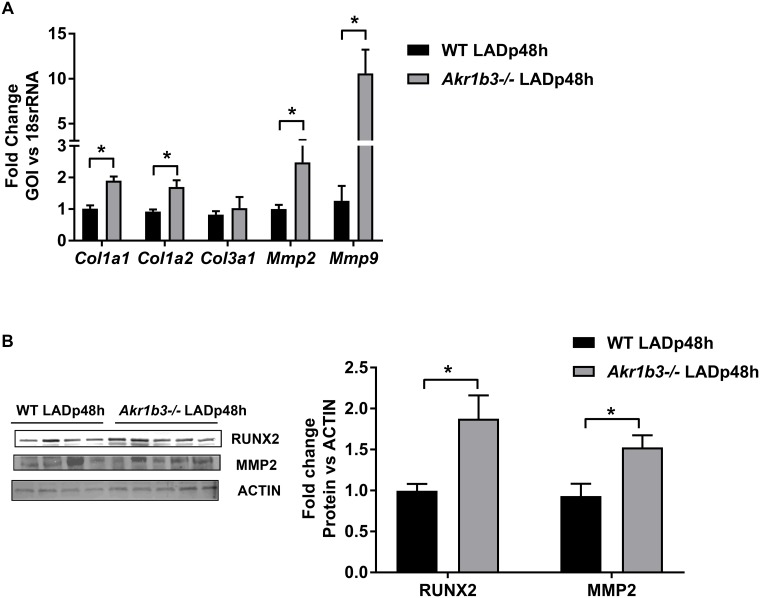
Increased expression of fibrotic factors in *Akr1b3* null I/R mice hearts. RNA isolated from heart tissues from mice subjected to I/R. (A) qRT-PCR on collagens (*Col1a1*, *Col1a2*, *Col3a1)* and MMPs (*Mmp2* and *Mmp9)*, N = 5 mice/group. (B) Western blot analysis on RUNX2 and MMP2 expression in heart tissues subjected to I/R. N = 4/group. Error bars represent mean ± SEM. * p<0.05, unless otherwise noted.

Activated fibroblasts are the primary source of increased extracellular matrix, which contributes to tissue fibrosis [35, 36]. The extracellular matrix of myocardium is largely composed of structural proteins, type I and III collagens, which not only provide mechanical support but also force for contraction. The homeostasis of collagens is maintained by the delicate balance between their synthesis and degradation via matrix metalloproteinases (MMPs). The β- catenin pathway has been linked to induction of genes encoding collagens and MMPs [37–39]. We next examined if collagens and MMPs were altered in mice hearts devoid of *Akr1b3* post- I/R. qRT-PCR analysis on Type I and III collagens (*Col1a1*, *Col1a2*, *Col3a1*) and MMPs (*Mmp2* and *Mmp9*) revealed an increase in both types of collagens as well as MMPs in mice hearts devoid of *Akr1b3* after I/R (Fig 4A). Further, we observed increases in RUNX2 and MMP2 protein expression in *Akr1b3* null mice hearts (Fig 4B). These results suggest that in mice hearts devoid of *Akr1b3*, increased expression of collagens and MMPs may contribute to remodeling responses after I/R.

### Lack of changes in fibrotic genes at 28 days post infarction

**Fig 5:**
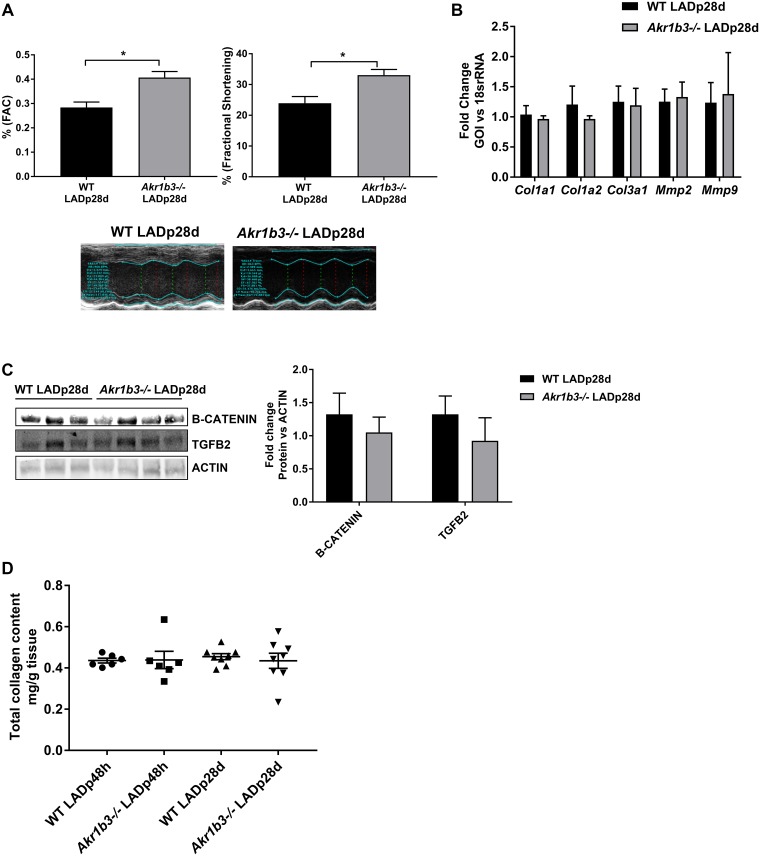
Absence of changes in expression of fibrotic factors in *Akr1b3* null I/R mice hearts after 28 days of recovery. Mice were subjected to LAD and studies were done 28 days post infarction recovery. (A) Echocardiographic measurements made in WT and *Akr1b3* null mice. Functional measurements, fractional shortening (FS) and fractional area change (FAC) are expressed as %, with representative image, N = 10 mice/group. (B) qRT-PCR on collagens (*Col1a1*, *Col1a2*, *Col3a1)* and MMPs (*Mmp2* and *Mmp9)* from heart tissue, N = 5 mice/group. (C) Western blot from heart lysates for detection of β-catenin and TGFB2 normalized to Beta-Actin, N = 4 mice/group. (D) Total collagen levels in hearts from mice subjected to LAD post 48 h and 28 days of recovery, N = 4 mice/group. Error bars represent mean ± SEM. * p<0.05, unless otherwise noted.

Post-infarction remodeling is a complex process in which cardiomyocyte loss is accompanied by cellular hypertrophy and fibrosis. Initially an adaptive response, extended cardiac fibrosis can lead to maladaptive remodeling and heart failure. Our results indicate that there was no evidence of hypertrophy in either WT or *Akr1b3* null mice hearts, but that there was induction of genes linked to fibrotic remodeling in mice hearts devoid of *Akr1b3*. If ablation of *Akr1b3* results in fibroblast activation and induction of genes linked to fibrotic remodeling, we sought to test whether this activation and induction persisted even after a month of infarction, without culminating in fibrosis. Hence we assessed echocardiographic measurements on WT and *Akr1b3* null mice 28 days post-LAD occlusion/recovery. We found a significant increase in fractional shortening and fractional area change measurements in *Akr1b3* null LAD mice compared to WT, which is consistent with overall superior cardiac function in the hearts devoid of *Akr1b3* (Fig 5A). Further, RNA analysis on fibrotic marker expression failed to show any differences between WT and the *Akr1b3* null mice on day 28 post-LAD (Fig 5B). Further, β-catenin and TGFB2 protein levels were comparable between both groups of mice at day 28 post-LAD (Fig 5C). We measured the total collagen levels in heart tissues 48 hrs and 28 days post LAD in both WT and mice devoid of *Akr1b3* and found no significant differences in collagen levels (Fig 5D). Taken together, these results indicate that changes in β-catenin, TGFB2 and fibrotic markers were unique to early phase of recovery (48 hours) in *Akr1b3* mice, and not sustained at the later phase of recovery (28 days).

### β-catenin inhibition ablates the beneficial effects of deletion of *Akr1b3* on expression of mesenchymal markers in the heart in I/R

**Fig 6:**
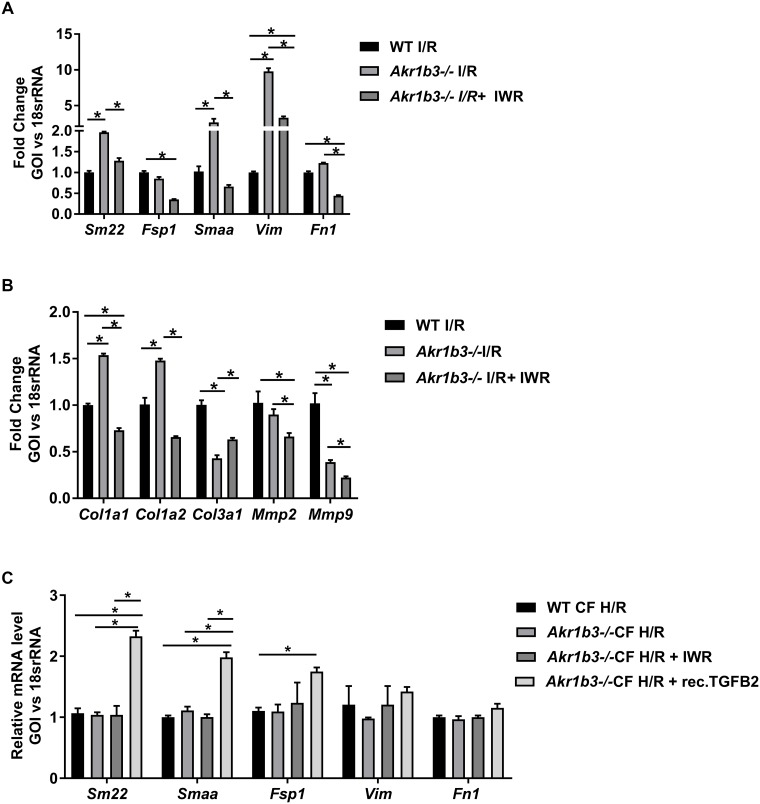
β-Catenin pathway inhibitor reverses the *Akr1b3* null mediated protection and mesenchymal marker expression. Mice hearts were subjected to *ex vivo* I/R using Langendorff technique. (A) qRT-PCR on heart tissues for mesenchymal markers as indicated, N = 3. (B) qRT-PCR on heart tissues for collagen and matrix metalloproteinases as indicated, N = 3 mice/group. (C) qRT-PCR on primary CF for mesenchymal markers treated either with recombinant TGFB2 or IWR as indicated, N = 5/group. Error bars represent mean ± SEM. * p<0.05, unless otherwise noted.

To confirm the mesenchymal activation observed in mice devoid of *Akr1b3* was due to enhanced β-catenin levels, we employed IWR-endo, an inhibitor of Wnt-β catenin pathway [40]. Using an *ex-vivo* isolated perfused heart for model of I/R, *Akr1b3* null mice hearts were treated with either vehicle or IWR-endo during the 30 min of ischemia and 60 min of reperfusion. qRT-PCR analysis from the RNA extracted from heart tissues revealed that IWR-endo treatment reduced the expression of mesenchymal and fibrotic markers after I/R in these mice hearts when compared to the vehicle treatment (Fig 6A and 6B). Further, to determine if cardiac fibroblasts (CF) at key site for the observed mesenchymal markers changes, primary CF were isolated from WT and *Akr1b3* null mice and subjected to H/R in the presence of recombinant TGFB2 protein. Treatment of IWR in CFs was used to establish the link to β-catenin. Data presented in Fig 6C shows no significant changes in mesenchymal markers in WT and *Akr1b3-/-* CF, while treatment with TGFB2 protein upregulated the mesenchymal markers. IWR treatment did not lead to activation of mesenchymal markers in WT and *Akr1b3-/-* CFs (Fig 6C). These results reveal that mesenchymal activation in *Akr1b3-/-*, is in part, via TGFB2 in CFs.

## Discussion

Post infarct remodeling in the heart is a complex process, where I/R-associated necrosis and apoptosis of cardiomyocytes is compensated by processes such as cardiomyocyte hypertrophy, transdifferentiation of endothelial to mesenchymal cells (EndMT) and fibroblast activation. *Akr1b3* null mice failed to show any hypertrophy. Our studies revealed that enhanced β-catenin signaling at an early time point of reperfusion in mice devoid of *Akr1b3* was beneficial to hearts that had been subjected to I/R. The increased TGFB2 and β-catenin protein suggested the EndMT pathway as a potential mechanism. However, this possibility was ruled out as endothelial markers were not downregulated.

*Akr1b3* null mice hearts displayed enhanced expression of mesenchymal markers, suggestive of fibroblast activation. Several studies had suggested the role of β-catenin in fibrosis and mesenchymal activation as a key driver of wound healing [41–44]. Published studies have shown that I/R can induce formation of epicardium-derived cells, which then differentiate into mesenchymal cells expressing fibroblast and smooth muscle cell markers [45, 46]. Consistent with these findings, our data show that the mesenchymal markers are upregulated during I/R in the *Akr1b3* null hearts and that these increases require β-catenin.

Activation of β-catenin in the mice hearts devoid of *Akr1b3* during I/R in our study could be attributed to either TGFB2 or GSK3B phosphorylation or both. Studies have shown that activation of β-catenin could be due, in part, to phosphorylation of GSK3β. The phosphorylation of GSK3β by p-Akt destabilizes GSK3β complex, resulting in the dissociation of β-catenin from the GSK3β complex. This process leads to nuclear translocation of β-catenin [26, 47]. Earlier studies in rats have shown that the cardioprotection afforded by phyllanthus emblica was linked to increasing levels of p-Akt and β-catenin, as well as increased GSK3β phosphorylation [25, 27, 48]. Since our earlier studies demonstrated reduced Tyr216 GSK3β phosphorylation during I/R in *Akr1b3-/-* hearts compared to WT hearts (14), here we focused on the role of TGFB2. Since TGFB2 has been implicated in β-catenin activation and cellular plasticity [21, 30], it is conceivable that activation of β-catenin in hearts devoid of *Akr1b3* could also be due to increases in TGFB2. Our studies in cardiac fibroblasts from WT and *Akr1b3* null mice subjected to H/R revealed only modest activation of mesenchymal markers in the presence of TGFB2, indicating other mechanisms are also contributing to mesenchymal activation. Specifically, in addition to TGFB2, secreted factors from other cells in heart may play an important role in activation of mesenchymal markers in our study.

The impact of mesenchymal activation on cardiac remodeling post infarction may, in part, explain reduced I/R injury in *Akr1b3-/-* hearts. Repair mechanisms to manage tissue scaring are essential for viable recovery after I/R. We believe β-catenin related changes may be of help in reducing the tissue scar through mesenchymal activation. In our studies we show that CF activation results in upregulation of both matrix metalloproteinases and collagens in *Akr1b3-/-* hearts, key players that facilitate removal and replacement of necrotic tissue. Hence we posit, in *Akr1b3-/-* mice, TGFB2 mediated mesenchymal activation may aid in the clearance of necrotic tissue thereby reducing injury.

MMPs are involved in physiological as well as pathological processes, such as inflammation, tumor metastasis and tissue remodeling [49]. Several studies have shown that activated MMPs mediate injury to contractile apparatus as well as that they affect the structural proteins within the intra- and extracellular matrix lattices [50–52]. Hence, inhibition of MMPs is considered as a therapeutic strategy to improve functional recovery in the heart after I/R [53]. Though isoform-specific *Mmp* knockout (global) mice showed cardioprotection, inhibitor studies as well as macrophage-specific MMP9 overexpression studies contradicted the earlier observations [54, 55]. Such studies underscore the complexity and cell type-specificity of MMP actions, both in development and in I/R in the adult heart. In this context, our studies demonstrated acute increases in *Mmp2*, *Mmp9*, as well as collagenases, without increases in the total collagen content in the mice hearts devoid of *Akr1b3* in I/R. Hence our findings on MMP changes are those in line with macrophage specific MMP9 overexpression mice and MMP9 inhibitor studies [54, 55].

Several studies point out the importance of prolonged structural alteration resulting in fibrosis and heart failure. TGFB2 and β-catenin mediated changes in our studies are likely related to acute structural alterations. Studies in the literature are mixed in regard to the role of TGFB in I/R hearts, with some studies showing the detrimental effects of TGFB and others showing cardioprotective effects of TGFB during I/R. [56–60] Likewise, studies in β-catenin were also contradictory, regarding its role in I/R hearts. Nevertheless, several studies have underscored the importance of β-catenin activation in reducing I/R injury either by reducing oxidative stress or apoptosis [41–44]. We observed that the cardioprotective effects in *Akr1b3* null mice in acute (48 hours) as well as extended periods of recovery (28 days) was linked to β-catenin changes. Our study identifies that β-catenin-mediated mesenchymal activation is linked to reduced myocardial I/R injury and that this pathway is downstream of the AKR1B3 pathway. In myocardial I/R, deletion of *Akr1b3* modulates the myocardial β-catenin pathway and consequent induction of mesenchymal markers as well as genes linked to fibrotic remodeling, thereby highlighting inhibition of the AKR1B3 pathway as a key strategy in protection against myocardial injury after infarction.

## Materials and methods

### Animals used

All animal experimentations were performed with the approval of the Institutional Animal Care and Use Committee at New York University School of Medicine. Male *Akr1b3* null mice- and wild type littermate mice were used as described earlier [8, 61]. Surgical procedures related to coronary artery ligation (LAD) were performed as previously described [8]. Briefly male mice aged approximately 4 months of age were anesthetized and subjected to LAD/reperfusion. LAD was ligated for 30 min and then blood flow was restored. Mice were allowed to recover and measurements were made after 48 h or 28 days of recovery. For functional measurements, mice were anesthetized with isoflurane via a nose cone and 2-dimensional echocardiography was performed on a Vevo 2100 System (Visual Sonics, Ontario, Canada). The left ventricular end-diastolic and end-systolic dimensions were measured and percent fractional shortening was calculated. Echocardiographic measurements were made before the surgery and after 48 h or 28 days of recovery. Age and sex matched controls were used. Cohort of hearts were used to assess area at risk and infarct area by 2,3,5-triphenyl-2H-tetrazolium chloride and Evan’s blue staining as published earlier [62, 63].

### *Ex-vivo* I/R

Experiments were carried out as described earlier [8, 15]. Briefly, mice were anesthetized using ketamine/xylazine. The hearts were rapidly excised and perfused through the aorta in a non-recirculating mode, using an isovolumic perfusion system through Langerndorff technique (LT) with Krebs-Henseleit buffer. Perfusion pO_2_>600 mm Hg was maintained in the oxygenation buffer. IWR-endo was added at 10μM final concentration to the buffer, whereas DMSO was added for vehicle control. Hearts were perfused with IWR or vehicle starting at 10 min prior to initiation of ischemia for 30 min and continued throughout 60 min of reperfusion.

Lactate dehydrogenase (LDH) levels in plasma (*in vivo*) and effluents (*ex vivo*) were measured using a commercially available kit (Pointe Scientific, Canton, MI).

### Cell culture

For transfection in mouse aortic endothelial cells, siRNA specific against *Akr1b3* was obtained from Life Technologies and were transfected using electroporation kit obtained from Lonza. 48 h post transfection experiments were conducted. For H/R experiment, cells were placed in a hypoxia chamber (Biospherix, Lacona, NY) for 30 min of hypoxia (0.5% O_2_, 5% CO_2_) at 37°C followed by 60 min of reoxygenation in 5% CO_2_ incubator (hypoxia/reoxygenation, H/R). Cells were collected at the end of reoxygenation in ice cold PBS for further analysis.

### Primary cardiac fibroblast isolation

Murine primary cardiac fibroblasts were isolated using the protocol mentioned [64]. Briefly, mince the hearts in ice-cold PBS into a size of 1mm using scalpel. The minced tissue was digested using digestion buffer (100U/ml collagenase II, 0.1% trypsin in HBSS buffer). Cells isolated were then plated in fibroblast medium (DMEM/F12, 10%FBS, 100U/ml Pen-Strep, 1X Glutamine, 100uM Ascorbic acid). Recombinant TGFB2 was purchased from R &D systems and used at a concentration of 10ng/ml.

### Collagen measurements

Total collagen levels from heart tissues subjected to LAD post 48hrs and 28 days were measured using total collagen assay kit (Biovison). Briefly, tissues were homogenized in concentrated HCl and hydrolyzed at 120°C for 3 h. Hydrolyzed sample were vacuum evaporated and subjected to quantification as per manufacturer‘s protocol.

### Western blot analysis

Total lysates from heart tissues or cells were prepared using lysis buffer (Cell Signaling). Tissues/cells were crushed using beads for homogenization and were quantified. For the detection of the protein following antibodies were used β-catenin, RUNX2 (cell signaling), TGFB2 (Santacruz), MMP2 (abcam), β-ACTIN (sigma) and ARKR1B3 (GeneTex). Antibodies were used at a final concentration of 1μg/ml.

### Realtime PCR

cDNA was prepared from whole heart tissues or cells using RNAeasy isolation kit (Qiagen). cDNA was prepared using iScript cDNA synthesis kit (BioRad). Fast sybrgreen mastermix and Taqman probes were used for quantification and data were normalized using 18s rRNA. Details of the primers used are in S1 Table.

### Statistics

All values are presented as the mean ± standard error of the mean. Data were analyzed by unpaired two-tailed t-tests to assess the difference between groups as specified. A probability value of ≤ 0.05 was considered significant.

## Supporting information

S1 Table Primers used. (PDF)
