# Supplementary material for: Aldose reductase modulates acute activation of mesenchymal markers via the β-catenin pathway during cardiac ischemia-reperfusion
Source: PLoS One. 2017 Nov 30;12(11):e0188981. doi: 10.1371/journal.pone.0188981 (PMC5708684; doi:10.1371/journal.pone.0188981)
Supplement: S1 Table — (PDF) [file pone.0188981.s001.pdf]

Taqman probes used:

Tnfa	MM00443258
Tgfb1	MM01178820
Tgfb2	MM01321738
Akr1b3	MM03047802
Sm22	MM00441661
Mmp9	MM00442991
Mmp2	MM00439498
18s	X03205.1

Sybrgreen primers used:

	**Forward**	**Reverse**
Snai1	AGCGGTCAGCAAAAGCAC	CTTGTGTCTGCACGACCTGT
Snai2	GATGTGCCCTCAGGTTTGAT	ATCTGTGGCAAGGCTTTCTC
Fsp1	AACTTGTCACCCTCTTTGCC	TCAGCACTTCCTCTCTCTTGG
Smaa	TTAGGGTTCAGTGGTGCCTC	ATCACCAACTGGGACGACAT
Vim	GGATTCCACTTTCCGTTCAA	GAAATTGCAGGAGGAGATGC
Fn1	TTGGTGATGTGTGAAGGCTC	ACCTCTGCAGACCTACCCAG
Cdh5	TCTTGCCAGCAAACTCTCCT	TTGGAATCAAATGCACATCG
Pecam1	TCCGTGGTAGCAGAAGTCAA	CGGTACAGGAGAATGCAGGT
Cldn5	GTGAGACCAGGTCCAGGCTA	CTGCTAACCTGAAAGGGCAG
Col1a2	CAGGTCCTTGGAAACCTTGA	CTGGGAACTTTGCTGCTCA
Col1a1	GGTTTCCACGTCTCACCATT	CGGCTCCTGCTCCTCTTAG
Col3a1	TGGTTCTGGCTTCCAGACAT	CACCCTTCTTCATCCCACTC
